# Outpatient visits and secondary surgeries following open globe injuries: a single institution retrospective analysis

**DOI:** 10.1007/s10792-026-03991-2

**Published:** 2026-02-14

**Authors:** Rita Vought, Victoria Vought, Roger K. Henry, Marko Oydanich, Albert S. Khouri

**Affiliations:** Department of Ophthalmology and Visual Science, Rutgers New Jersey Medical School, Institute of Ophthalmology and Visual Science, 90 Bergen St, Suite 6100, Newark, NJ 07103 USA

**Keywords:** Open globe injury, Ocular injury, Secondary surgeries, Follow-up, Ocular trauma

## Abstract

**Purpose:**

Open globe injuries (OGIs) impose a significant burden on patients and the healthcare system. This study reports outpatient visits and secondary surgeries associated with OGIs

**Methods:**

A retrospective chart review of OGI repairs at a Level 1 Trauma Center from 2015-2023 was conducted. Two areas of resource utilization, outpatient office visits and secondary surgeries required within a year of the injury were recorded and predictors were identified

**Results:**

Of 619 patients (mean age 46±22 years; 76.7% male), most had OGIs from blunt traumatic etiology (57.0%) with injury in zone I (65.9%). On average, patients had 5.3±4.7 office visits, where 8% of patients had no follow-up, 76% had 1–9 visits, and 16% had 10 or more visits. Thirty-five percent required at least one secondary surgery (mean 0.5±0.8). Clinical factors, including presenting best-corrected visual acuity (BCVA), predicted utilization. Predictors for office visits included injury zone (*p*=0.02), retinal detachment (*p*<0.001), vitreous hemorrhage (*p*=0.014), and traumatic cataract (*p*=0.011). Retinal detachment (*p*<0.001), and traumatic cataract (*p*<0.001) were predictive of secondary surgeries. The most common surgeries were pars plana vitrectomy (n=124), cataract extraction (n=46), enucleation (n=33), and corneal transplant (n=21)

**Conclusion:**

OGI management often requires additional procedures with significant follow-up. Overall trends suggest greater utilization among eyes with significant injury that still maintain potential for visual recovery.

## Introduction

Open globe injuries (OGIs) are vision-threatening ocular emergencies that often lead to significant visual impairment or permanent vision loss [1]. They may result from ocular trauma or non-traumatic etiologies such as ocular surface disease and infection. The prognosis following OGI is driven by many factors including best-corrected visual acuity (BCVA) on presentation [2, 3]. Epidemiological analysis within the United States estimates an incidence rate of OGIs to be 4.49 per 100,000 individuals [4], although mechanisms range from assaults, recreational and work-related injuries among younger individuals and falls in elderly patients. While OGIs comprise a small portion of all ocular trauma, they are responsible for an outsized portion of both medical and economic burden. A 2020 study found that while 2.0% of ocular trauma cases were OGIs, they contributed to 9.4% of total costs associated with ocular trauma [4].

Limited literature has directly quantified healthcare resource utilization among OGI patients. For example, studies have discussed specific resources, such as follow-up visits, after OGIs [5] and general ocular trauma [6]. Although both sociodemographic and clinical risk factors for visual acuity outcomes have been thoroughly investigated [7–9], there has been little exploration into how these features may impact resource utilization at the patient level. Beyond the initial globe repair, patients may require additional procedures and extensive follow-up appointments to monitor and address complications [10]. These interventions place significant demands on the healthcare system. Many studies have attempted to quantify the cost burden of OGIs; however, this varies significantly based on geographical region and patient-based factors and may be less relevant to hospitals or patients with different characteristics [4, 5, 11]. Therefore, evaluation of OGI healthcare burden using generalizable variables irrespective of cost can be more appropriate to report the healthcare usage in this cohort, with the goal of providing findings that can be applied to a wider population.

This study identified two major areas of utilization to be quantified: outpatient office visits and secondary surgeries, defined as returns to the operating room excluding the initial OGI repair. These measures were chosen as variables that could be easily compared across institutions as a proxy for healthcare use, while reducing the impact of less clinically relevant patient-specific features such as insurance coverage and location. Therefore, the objective of this study was to characterize these measures of resource utilization based on presenting clinical features in a diverse patient population undergoing open globe repairs at a Level I Trauma Center.

## Methods

### Data collection

A retrospective review of OGI repairs was conducted at a Level 1 Trauma Center in Newark, NJ, covering cases from January 2015 to August 2023. Data were obtained from individually reviewing de-identified patient records from the Epic Electronic Health Record (EHR). Repairs performed within one year of the data collection period (August 2024) were excluded. In addition, any OGIs that were non-operable were also excluded as they would not have been queried as a ruptured globe repair.

Demographic data extracted included age at time of globe repair, sex, race and ethnicity, and insurance type [12]. Clinical characteristics of the injury were recorded: presence of trauma; type of trauma (blunt or penetrating); zone of injury; and BCVA at presentation; The presence of the following was also noted: uveal prolapse, surgical wound dehiscence, endophthalmitis, intraocular foreign body, retinal detachment, vitreous hemorrhage, traumatic cataract, orbital floor fracture, and lid laceration. Age was stratified into three cohorts: 0–17, 18–64, or 65 and older. Visual acuities were grouped as follows: no light perception (NLP), light perception (LP) or hand motion (HM), counting fingers (CF) or 20/400 to 20/200, 20/150-20/50, 20/40 or better, or unable to assess as documented in the EHR.

Imaging studies of any structures within the head and neck within one year of the OGI were noted. The outcomes of interest to represent areas of resource utilization included the total number of outpatient office visits and secondary surgeries. “Secondary surgeries” encompassed any return to the operating room within one year of the OGI for an ophthalmic procedure, excluding the initial globe repair. Multivariable logistic regressions were used to determine associations clinical characteristics versus outcome measures. Associations between clinical factors and common secondary surgeries were evaluated. Data analysis was performed using IBM SPSS Statistics (v28).

### Ethics approval

This study adhered to the Declaration of Helsinki. Institutional Review Board approval was obtained through Rutgers, New Jersey Medical School (IRB Pro2019001861).

## Results

### Patient demographics and clinical features

Between 2015 and 2023, 619 primary OGI repairs (mean age 46±22 years; 76.7% male) were performed at University Hospital in Newark, NJ. Patients mainly identified as Hispanic Other Race (32%), non-Hispanic Black (28.4%), and non-Hispanic White (27.1%). Most individuals had private insurance (49.3%), Medicare (17.6%) or no insurance coverage (16.0%) (Table 1).

**Table 1:** Sociodemographic descripion of individuals with open globe injuries up to one year after the injury (n=619)

	Total	
	n	%
Overall	619	100
*Age*		
0–17.9	55	8.9
18–64.9	430	69.5
65+	134	21.6
*Sex*		
Male	475	76.7
Female	144	23.3
*Race/Ethnicity*		
Black, non-Hispanic	176	28.4
Black, Hispanic	2	0.3
White, non-Hispanic	168	27.1
White, Hispanic	4	0.6
Other, non-Hispanic	65	10.5
Other, Hispanic	198	32.0
Unknown	6	1.0
*Insurance status*		
Private insurance	305	49.3
Medicare	109	17.6
Medicaid	28	4.5
Worker’s Comp	62	10.0
Other insurance	16	2.6
No insurance	99	16.0

Presenting clinical features of subjects were classified and delineated in Table 2. The right eye was involved in 46.4% of OGIs. The most common etiology was traumatic (98.1%), and 57.0% of traumatic OGIs were blunt. A small portion (1.9%) were nontraumatic, which included spontaneous perforation secondary to corneal ulcers and ocular surface disease. Most patients presented with a BCVA of LP/HM (45.2%) or NLP (18.2%). Sixty-six percent of injuries were in zone I. Uveal prolapse was present in 66.4% of cases. Surgical wound dehiscence, traumatic cataract, eyelid laceration, intraocular foreign body, vitreous hemorrhage, orbital floor fracture, retinal detachment, and endophthalmitis were noted in 16.6, 15.3, 15.0, 12.1, 12.1, 11.3, 7.8, and 1.3%, respectively. The most common imaging studies obtained over the course of a year were B-scans (n=881), Computed tomography (CT) orbit or CT maxillofacial without contrast (n=509), CT head without contrast (n=249), and Optical Coherence Tomography (OCT) retina (n=177).

**Table 2:** Clinical factors impacting the number of office visits and secondary surgeries required by individuals with open globe injuries up to one year after the injury

		Total		Office visits		Secondary surgeries	
		n	%	Mean (SD)	Range	Mean (SD)	Range
*Traumatic injury*							
	Yes	607	98.1	5.3 (4.7)	0–27	0.5 (0.8)	0–4
	No	12	1.9	4.8 (3.6)	1–13	0.8 (1.0)	0–3
	*p-value*			0.38		0.037	
*Type of trauma*							
	Blunt	346	55.9	4.8 (4.5)	0–27	0.4 (0.8)	0-4
	Penetrating	261	42.2	5.9 (4.9)	0–27	0.6 (0.8)	0-4
	Non-traumatic	12	1.9	4.8 (3.6)	1–13	0.8 (1.0)	0-3
	*p-value*			0.049		0.13	
*Presenting best-corrected visual acuity*							
	NLP	113	18.2	3.7 (3.4)	0–18	0.4 (0.6)	0–2
	LP/HM	280	45.2	5.9 (5.1)	0–27	0.7 (0.9)	0–4
	CF-20/200	105	17	6.5 (5.4)	0–27	0.5 (0.8)	0–4
	20/150-20/50	65	10.5	5.0 (3.1)	1–13	0.3 (0.6)	0–3
	≥20/40	46	7.4	3.5 (2.6)	0–10	0.1 (0.3)	0–1
	Unable	10	1.6	2.5 (1.5)	0-5	0.2 (0.4)	0-1
	*p-value*			0.20		<0.001	
*Zone of injury*							
	I	408	65.9	5.7 (4.9)	0-27	0.5 (0.8)	0-4
	II	142	22.9	4.4 (3.8)	0-22	0.4 (0.8)	0-4
	III	69	11.2	4.6 (4.4)	0-28	0.5 (0.7)	0-3
	*p-value*			0.02		0.11	
*Uveal prolapse*							
	Yes	411	66.4	5.3 (4.6)	0-27	0.5 (0.8)	0-4
	No	208	33.6	5.3 (4.9)	0-27	0.5 (0.8)	0-4
	*p-value*			0.80		0.97	
*Surgical wound dehiscence*							
	Yes	103	16.6	5.4 (5.9)	0-27	0.4 (0.8)	0-4
	No	516	83.4	5.3 (4.4)	0-27	0.5 (0.8)	0-4
	*p-value*			0.29		0.99	
*Endophthalmitis*							
	Yes	8	1.3	7.9 (4.2)	2-13	1.1 (1.4)	0-3
	No	611	98.7	5.3 (4.7)	0-27	0.5 (0.8)	0-4
	*p-value*			0.27		0.098	
*Retinal detachment*							
	Yes	48	7.8	8.2 (5.1)	0-22	1.1 (1.0)	0-4
	No	571	92.2	5.0 (4.6)	0-27	0.5 (0.7)	0-4
	*p-value*			<0.001		<0.001	
*Intraocular foreign body*							
	Yes	75	12.1	6.3 (4.8)	0-27	0.6 (0.9)	0-4
	No	544	87.9	5.2 (4.6)	0-22	0.5 (0.8)	0-4
	*p-value*			0.23		0.097	
*Vitreous hemorrhage*							
	Yes	75	12.1	6.9 (5.7)	0-27	0.7 (0.9)	0-4
	No	544	87.9	5.1 (4.5)	0-27	0.5 (0.8)	0-4
	*p-value*			0.014		0.63	
*Orbital floor fracture*							
	Yes	70	11.3	4.7 (4.3)	0-18	0.6 (0.7)	0-3
	No	549	88.7	5.4 (4.7)	0-27	0.5 (0.8)	0-4
	*p-value*			0.41		0.51	
*Traumatic cataract*							
	Yes	95	15.3	6.8 (4.4)	0-27	0.8 (0.8)	0-3
	No	524	84.7	5.0 (4.7)	0-27	0.4 (0.8)	0-4
	*p-value*			0.011		<0.001	
*Eyelid laceration*							
	Yes	93	15	5.7 (4.7)	0-22	0.7 (0.8)	0-4
	No	526	85	5.2 (4.7)	0-27	0.5 (0.8)	0-4
	*p-value*			0.25		0.070	

### Outpatient visits and secondary surgeries

**Fig. 1:**
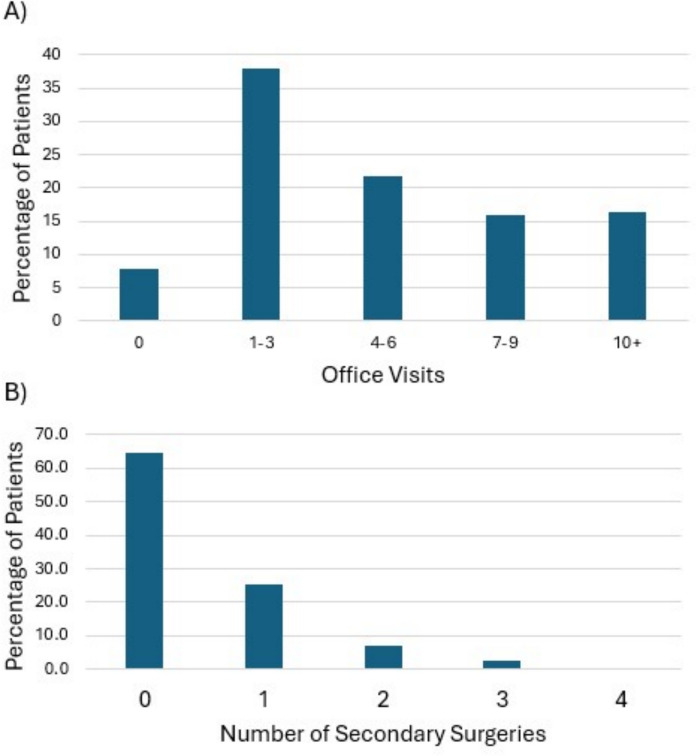
Distribution of patients by the number of office visits (A) and secondary surgeries (B) within one year

Overall, subjects had a mean 5.3±4.7 outpatient visits and 0.5±0.8 secondary surgeries within a year from the date of presentation. Eight percent (n=49) of patients had no follow-up, 76% had between 1-9 visits (n=468), and 16% (n=102) had 10 or more visits (Fig. 1A). Of all patients, 64.5% (n=399) had no secondary surgeries, 25.2% (n=156) had one, and 10.3% (n=64) had two or more (Fig. 1B).

**Fig. 2:**
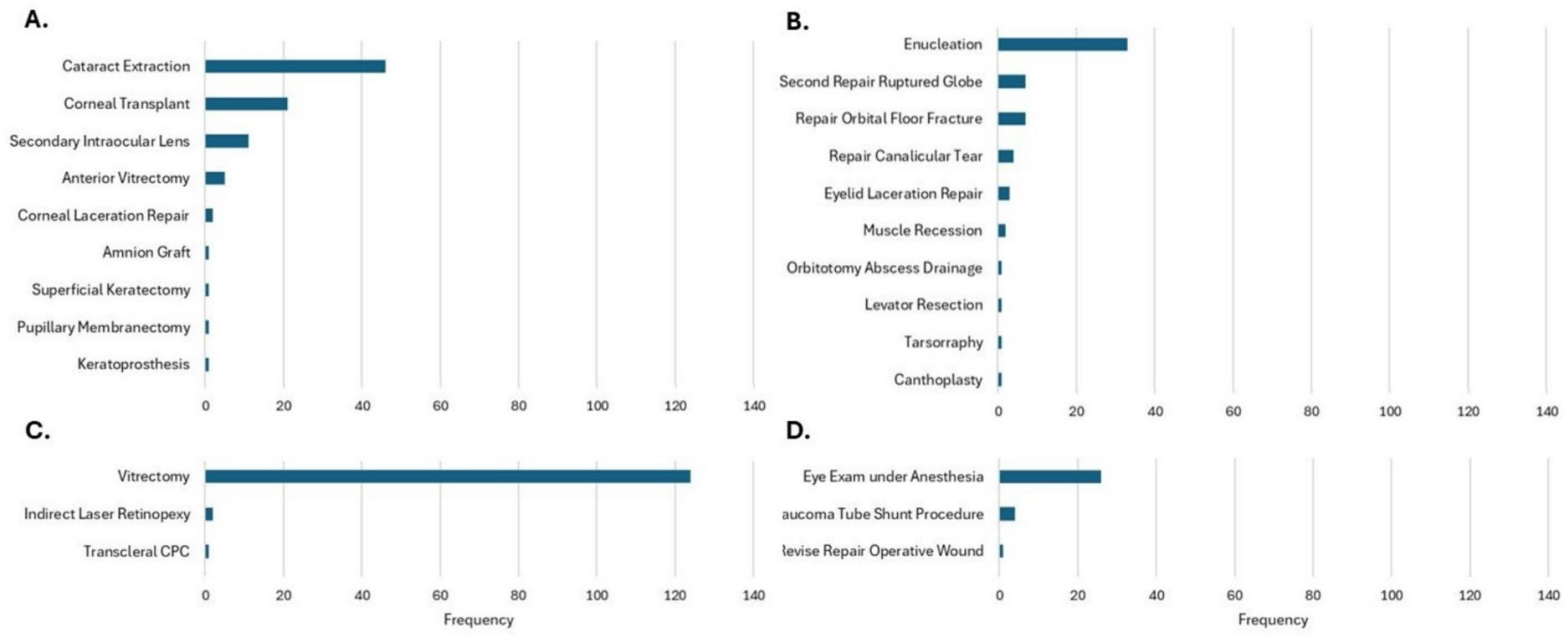
Frequency of secondary OR surgeries (n=307) performed within one year after OGI repair stratified by **A** Anterior Segment Surgeries, **B** Orbit and Adnexal Surgeries, **C** Posterior Segment Surgeries, and **D** Other Procedures

Multiple regression analysis evaluated the impact of clinical factors in predicting office visits (*p*<0.001) and secondary surgeries (*p*<0.001). Table 2 delineates the statistical significance of clinical covariates as predictors for these resources. The presence of retinal detachment and traumatic cataract were predictive for both outpatient visits and secondary surgeries. The most common secondary surgeries (Fig. 2) were pars plana vitrectomy (124 procedures on 15.0% of patients), cataract extraction (46 procedures on 7.4% of patients), enucleation (33 procedures, 5.3% of patients), and corneal transplant (21 procedures on 3.2% of patients). The predictive value of the same clinical variables for measures of the most common secondary surgeries is described in Table 3. BCVA was predictive for vitrectomy (*p*=0.004), enucleation (*p*<0.001), and corneal transplant (*p*=0.045). Endophthalmitis, retinal detachment, and vitreous hemorrhage were predictive for vitrectomy, while traumatic cataract was predictive for cataract surgery. Injury type, eyelid laceration, and OFF were predictive for enucleation. Injury type, surgical wound dehiscence, endophthalmitis, and retinal detachment were associated with corneal transplant.

**Table 3:** Association between clinical factors and common secondary surgeries

		Vitrectomy		Cataract extraction		Enucleation		Corneal transplant	
		Mean (SD)	Range	Mean (SD)	Range	Mean (SD)	Range	Mean (SD)	Range
Overall		0.2 (0.5)	0–3	0.1 (0.3)	0-1	0.1 (0.2)	0-1	0.0 (0.2)	0-2
*Traumatic injury*									
	Yes	0.2 (0.5)	0–3	0.1 (0.3)	0-1	0.1 (0.2)	0-1	0.0 (0.2)	0-1
	No	0.1 (0.3)	0–1	0.1 (0.3)	0-1	0.2 (0.4)	0-1	0.2 (0.4)	0-2
	*p-value*	0.93		0.2		0.47		<0.001	
*Type of trauma*									
	Blunt	0.2 (0.5)	0–3	0.0 (0.2)	0-1	0.1 (0.3)	1-1	0.0 (0.2)	0-2
	Penetrating	0.3 (0.6)	0–3	0.1 (0.3)	0–1	0.0 (0.1)	0–1	0.0 (0.2)	0–1
	Non–traumatic	0.1 (0.3)	0–1	0.1 (0.3)	0–1	0.2 (0.4)	0–1	0.2 (0.4)	0–1
	*p-value*	0.071		0.057		0.017		0.77	
*Presenting best-corrected visual acuity*									
	NLP	0.1 (0.3)	0–2	0.0 (0.0)	0–0	0.2 (0.4)	0–1	0.0 (0.1)	0–1
	LP/HM	0.3 (0.6)	0–3	0.1 (0.3)	0–1	0.0 (0.2)	0–1	0.1 (0.3)	0–2
	CF-20/200	0.2 (0.5)	0–2	0.2 (0.4)	0–1	0.0 (0.2)	0–1	0.0 (0.0)	0–0
	20/150-20/50	0.1 (0.4)	0–3	0.0 (0.2)	0–1	0.0 (0.0)	0–0	0.0 (0.0)	0–0
	≥20/40	0.0 (0.1)	0–1	0.0 (0.1)	0–1	0.0 (0.0)	0–0	0.0 (0.0)	0–0
	Unable	0.0 (0.0	0–0	0.0 (0.0)	0–0	0.0 (0.0)	0–0	0.0 (0.0)	0–0
	*p-value*	0.004		0.07		<0.001		0.045	
*Zone of injury*									
	I	0.2 (0.5)	0–3	0.1 (0.3)	0–1	0.0 (0.2)	0–1	0.1 (0.2)	0–2
	II	0.2 (0.5)	0–2	0.0 (0.1)	0–1	0.1 (0.3)	0–1	0.0 (0.1)	0–1
	III	0.2 (0.5)	0–2	0.0 (0.1)	0–1	0.1 (0.2)	0–1	0.0 (0.0)	0–0
	*p-value*	0.098		0.268		0.97		0.11	
*Uveal prolapse*									
	Yes	0.2 (0.5)	0–3	0.1 (0.3)	0–1	0.1 (0.2)	0–1	0.0 (0.2)	0–1
	No	0.2 (0.5)	0–3	0.1 (0.3)	0–1	0.0 (0.2)	0–1	0.1 (0.2)	0–2
	*p-value*	0.59		0.62		0.68		0.18	
*Surgical wound dehiscence*									
	Yes	0.2 (0.5)	0–2	0.0 (0.1)	0–1	0.0 (0.2)	0–1	0.1 (0.3)	0–2
	No	0.2 (0.5)	0–3	0.1 (0.3)	0–1	0.1 (0.2)	0–1	0.0 (0.1)	0–1
	*p-value*	0.354		0.55		0.130		<0.001	
*Endophthalmitis*									
	Yes	0.6 (0.7)	0–2	0.1 (0.4)	0–1	0.1 (0.4)	0–1	0.3 (0.5)	0–1
	No	0.2 (0.5)	0–3	0.1 (0.3)	0–1	0.1 (0.2)	0–1	0.0 (0.2)	0–2
	*p-value*	0.046		0.81		0.22		0.008	
*Retinal detachment*									
	Yes	0.7 (0.7)	0-2	0.1 (0.2)	0–1	0.0 (0.1)	0–1	0.1 (0.3)	0–1
	No	0.2 (0.5)	0-3	0.1 (0.3)	0–1	0.0 (0.2)	0–1	0.0 (0.2)	0–2
	*p-value*	<0.001		0.58		0.15		0.02	
*Intraocular foreign body*									
	Yes	0.3 (0.6)	0–3	0.1 (0.3)	0–1	0.0 (0.2)	0–1	0.0 (0.1)	0–1
	No	0.2 (0.5)	0–3	0.1 (0.2)	0–1	0.1 (0.2)	0–1	0.0 (0.2)	0–2
	*p-value*	0.42		0.41		0.50		0.76	
*Vitreous hemorrhage*									
	Yes	0.5 (0.7)	0–2	0.0 (0.2)	0–1	0.0 (0.2)	0–1	0.0 (0.1)	0–1
	No	0.2 (0.5)	0–3	0.1 (0.3)	0–1	0.1 (0.2)	0–1	0.0 (0.2)	0–2
	*p-value*	0.009		0.13		0.22		0.36	
*Orbital floor fracture*									
	Yes	0.2 (0.5)	0–2	0.0 (0.1)	0–1	0.2 (0.4)	0–1	0.0 (0.1)	0–1
	No	0.2 (0.5)	0–3	0.1 (0.3)	0–1	0.0 (0.2)	0–1	0.0 (0.2)	0–2
	*p-value*	0.94		0.7		0.005		0.80	
*Traumatic cataract*									
	Yes	0.2 (0.5)	0–2	0.4 (0.5)	0–1	0.0 (0.1)	0–1	0.0 (0.1)	0–1
	No	0.2 (0.5)	0–3	0.0 (0.1)	0–1	0.1 (0.2)	0–1	0.0 (0.2)	0–2
	*p-value*	0.54		<0.001		0.36		0.70	
*Eyelid laceration*									
	Yes	0.2 (0.5)	0–2	0.1 (0.2)	0–1	0.1 (0.3)	0–1	0.0 (0.1)	0–1
	No	0.2 (0.5)	0–3	0.1 (0.3)	0–1	0.0 (0.2)	0–1	0.0 (0.2)	0–2
	*p-value*	0.53		0.61		0.005		0.80	

## Discussion

This study is one of the largest single-institution reviews of OGIs, examining over 600 affected patients and analyzing the factors impacting the number of ophthalmic office visits and secondary surgeries within one year of the initial injury. These values have not been previously quantified and associated with injury characteristics in OGIs.

### Predictors of office visits and secondary surgeries

Assessment of clinical characteristics associated with greater healthcare use found that patients with poor presenting BCVA, such as LP/HM and CF or 20/400-20/200, had greater office visits and secondary surgeries. This trend held true in analysis of the common secondary surgeries excluding enucleation (Table 3). Although worse presenting VA is associated with poor final BCVA, other evaluations have found that BCVA typically stabilizes within the first three months of the initial injury, with a final BCVA improved about 3 lines of vision at one year [13]. Although the risk of significant visual impairment and legal blindness is high after OGI [13], any interventions that can preserve vision may significantly improve quality of life. Therefore, greater utilization of healthcare among patients with poor vision may reflect efforts to prevent progression to NLP. Similarly, zone of injury was a predictor for office visits, with the highest mean visits for zone I injuries (mean 5.7±4.9 vs 4.4±3.8 [zone II] vs 4.6±4.4 [zone III]). Zone I injuries have a better visual potential than zones II and III [14], likely due to decreased likelihood of posterior segment involvement. Although the structural integrity of the globe can be restored, damage to the retina and optic nerve may be irreparable [2, 15].

The type of trauma was also a predictor of office visits, with the most office visits for penetrating injuries (mean 5.9±4.9 [penetrating] vs 4.8±4.5 [blunt] vs 4.8±3.6 [non-traumatic]). Other examinations of OGIs found that penetrating injuries often have better prognosis than blunt injuries. Penetrating trauma tends to create a distinct entry wound with less risk of posterior segment involvement, while blunt trauma creates more complex ruptures with more risk of posterior segment involvement [16, 17]. Therefore, given higher potential for visual recovery in penetrating injuries, this etiology was also statistically predictive for more visits and secondary surgeries (Table 2). Conversely, eyes with poor prognosis had less utilization. For instance, BCVA was predictive of enucleation, with the highest mean among NLP patients (Table 3). This may be attributed to the lack of visual potential and risk of sympathetic ophthalmia. Patients presenting with BCVAs better than NLP may also undergo enucleations for deteriorating BCVA or other complications following OGI repair. Other predictors of enucleation included orbital floor fracture, eyelid laceration, and non-traumatic etiology. Among traumatic OGIs, orbital floor fractures and eyelid lacerations represent injury to the ocular adnexa. Both have been previously correlated with greater visual loss and the need for enucleation due to their association with corneoscleral injury and posterior globe involvement [15, 17, 18]. Non-traumatic OGIs comprised less than 2% of cases analyzed, which included cases of spontaneous perforations resulting from infectious or inflammatory conditions. Other examinations of non-traumatic OGIs have also associated these cases with similar causes [2]. Common etiologies such as infection, which may seed throughout the eye and cause rapid deterioration, may require aggressive intervention and eventual enucleation.

As expected, findings on presentation that are indicative for additional intervention, such as retinal detachment, vitreous hemorrhage, and traumatic cataracts, were also associated with more office visits [19]. Predictors for specific types of surgery were as anticipated (Table 3): endophthalmitis, retinal detachment, and vitreous hemorrhage were predictive for vitrectomy; presence of traumatic cataract was associated cataract extraction surgery; non-traumatic injury, surgical wound dehiscence, and endophthalmitis were predictive for corneal transplant. [19] The presence retinal detachment was statistically associated with need for corneal transplant although likely not clinically relevant. The type of injury was also a predictive factor for the total secondary surgeries, with the highest among non-traumatic OGIs (mean 0.8±1.0 vs 0.5±0.8). Due to the more chronic nature of these conditions (i.e. recurrent or nonhealing corneal ulcers), rather than an acute localized trauma, a longitudinal multi-faceted approach is required in the management of these patients.

### Study limitations

This study has limitations related to scope and the nature of the methodology. The sample focused only on OGI repairs, therefore excluding non-operable cases. In addition, since the data is derived from a single urban Level I Trauma Center in New Jersey, the patterns observed may not reflect those observed in rural settings, community hospitals, or other geographic regions. Because of the retrospective design, the completeness and accuracy of data were dependent on the documentation available on the Epic EHR. Data collection was performed using emergency department notes, ophthalmology consultation, outpatient, and inpatient notes, and operative reports to develop a comprehensive dataset and minimize inconsistencies. Additionally, the focus of this study was on ophthalmology-specific follow-up and secondary surgeries. In cases of polytrauma or other complex injuries, multiple specialties would have been involved in patient care, potentially affecting overall healthcare use. This multidisciplinary involvement was not included in this analysis, although it may reflect in the high number of imaging studies reported. Resource utilization was quantified based on the total office visits and surgeries documented within the Epic EHR. However, it is possible that some patients received ophthalmic follow-up care outside of our institution, such as with an ocularist after enucleation. This data may not have been captured in the dataset.

## Conclusion

This study provides valuable insight into the patterns of outpatient follow-up and secondary surgeries following OGI repairs in a large, diverse patient population over an extended time period. This snapshot of the first year following OGI for 619 patients found a significant burden of care represented by ongoing outpatient visits and secondary surgeries per patient (averaging more than 5 visits in a year and 35.5% patients requiring additional surgery). Presenting BCVA and type of trauma, both of which have been previously associated with visual prognosis, were found to be predictive of reported resources. Those with greater recovery potential despite severe injury had more office visits and secondary surgeries, reflecting treatment efforts by healthcare providers. Our findings are relevant for guiding future research that may quantify resource allocation using similar measures and identify areas for improvement. Future investigations may explore the role of multidisciplinary care in resource burden for patients with complex or polytraumatic injuries. Investigating other proxies of healthcare use, including care provided outside of a single institution, could further enhance the understanding of total resource usage.

## Data Availability

No datasets were generated or analysed during the current study.
